# Supplementary material for: HIVprotI: an integrated web based platform for prediction and design of HIV proteins inhibitors
Source: J Cheminform. 2018 Mar 9;10:12. doi: 10.1186/s13321-018-0266-y (PMC5845081; doi:10.1186/s13321-018-0266-y)
Supplement: Supplementary file 2 — Additional file 2. Source code of HIVProtI web server. [file 13321_2018_266_MOESM2_ESM.zip › HIVprotI_Source-code/hivprotI/weka-3-6-6/doc/weka/core/AdditionalMeasureProducer.html]

AdditionalMeasureProducer


---


|  |  |  |
| --- | --- | --- |
| **Overview** \| **Package** \| **Class** \| **Tree** \| **Deprecated** \| **Index** \| **Help** \| **Weka's home** | |  |
| **PREV CLASS**   **NEXT CLASS** | **FRAMES**    **NO FRAMES**     **All Classes** |  |
| SUMMARY: NESTED \| FIELD \| CONSTR \| METHOD | DETAIL: FIELD \| CONSTR \| METHOD |  |


---


## weka.core Interface AdditionalMeasureProducer

**All Known Implementing Classes:**: AdditiveRegression, ADTree, AttributeSelectedClassifier, AveragingResultProducer, Bagging, BallTree, BayesNet, BayesNetGenerator, BFTree, BIFReader, ClassifierSplitEvaluator, CostSensitiveClassifierSplitEvaluator, CoverTree, CrossValidationResultProducer, DatabaseResultProducer, DecisionTable, DensityBasedClustererSplitEvaluator, DTNB, EditableBayesNet, FT, GridSearch, IBk, J48, J48graft, JRip, KDTree, LADTree, LearningRateResultProducer, LinearNNSearch, LMT, M5Base, M5P, M5Rules, NBTree, NearestNeighbourSearch, PART, PerformanceStats, RandomForest, RandomSplitResultProducer, RegressionSplitEvaluator, REPTree, Ridor, SimpleCart, SimpleLogistic, SMOreg, TreePerformanceStats

---

``` public interface AdditionalMeasureProducer ```

Interface to something that can produce measures other than those
calculated by evaluation modules.

**Version:**
:   $Revision: 1.8 $

**Author:**
:   Mark Hall (mhall@cs.waikato.ac.nz)

---

| **Method Summary** | |
| --- | --- |
| `java.util.Enumeration` | `enumerateMeasures()`             Returns an enumeration of the measure names. |
| `double` | `getMeasure(java.lang.String measureName)`             Returns the value of the named measure |

**Method Detail**

### enumerateMeasures

```
java.util.Enumeration enumerateMeasures()
```

:   Returns an enumeration of the measure names. Additional measures
    must follow the naming convention of starting with "measure", eg.
    double measureBlah()

    :   **Returns:**: an enumeration of the measure names

---


### getMeasure

```
double getMeasure(java.lang.String measureName)
```

:   Returns the value of the named measure

    :   **Parameters:**: `measureName` - the name of the measure to query for its value **Returns:**: the value of the named measure **Throws:**: `java.lang.IllegalArgumentException` - if the named measure is not supported


---


|  |  |  |
| --- | --- | --- |
| **Overview** \| **Package** \| **Class** \| **Tree** \| **Deprecated** \| **Index** \| **Help** \| **Weka's home** | |  |
| **PREV CLASS**   **NEXT CLASS** | **FRAMES**    **NO FRAMES**     **All Classes** |  |
| SUMMARY: NESTED \| FIELD \| CONSTR \| METHOD | DETAIL: FIELD \| CONSTR \| METHOD |  |


---
